# A commentary on ‘Silent gallbladder stone in kidney transplantation recipients: should it be treated? A retrospective cohort study’

**DOI:** 10.1097/JS9.0000000000001513

**Published:** 2024-05-03

**Authors:** Fangxiao Xia, Chao Su

**Affiliations:** a Department of Nephrology, Ganzhou Hospital of Guangdong Provincial People’s Hospital, Ganzhou Municipal Hospital, Ganzhou, People’s Republic of China; b Discipline of Physiology, School of Medicine, University of Galway, Galway, Ireland

*Dear Editor,*

A recent study^1^ investigated the role of pre-KT cholecystectomy in preventing biliary and surgical complications. While the study addresses an important clinical question concerning the management of silent gallbladder stones in patients before kidney transplantation (KT), several methodological limitations undermine the robustness of its conclusions.

Firstly, the retrospective design of the study introduces inherent biases and limitations. Retrospective studies are susceptible to selection bias, confounding, and information bias, which can compromise the validity of the results. A prospective cohort or randomized controlled trial would have provided stronger evidence to support the conclusions drawn in the article. Secondly, the sample size, particularly for certain subgroups, such as KT recipients who underwent pre-KT cholecystectomy, is relatively small. Small sample sizes reduce the statistical power of the study and increase the likelihood of type II errors, limiting the generalizability and reliability of the findings.

Additionally, the absence of a control group further complicates the interpretation of the results and limits the ability to draw causal inferences. Furthermore, the study fails to adequately address potential confounding factors that could influence the outcomes of interest. Factors such as comorbidities, concomitant medications, and surgical techniques may confound the association between pre-KT cholecystectomy and biliary complications. Failure to adjust for these confounders may lead to biased estimates of the treatment effect. Moreover, the conclusion that preemptive cholecystectomy for asymptomatic cholecystolithiasis may reduce surgical risk in KT candidates is extrapolated from the study findings without considering alternative management strategies or potential harms associated with surgery. The decision to undergo cholecystectomy should be based on a comprehensive assessment of the risks and benefits, taking into account patient preferences, comorbidities, and surgical expertise. In light of these limitations, I recommend that the authors conduct further research using a prospective study design with a larger sample size and appropriate control groups to validate their findings. Additionally, a more thorough analysis accounting for potential confounders and a nuanced discussion of the implications of the study results would strengthen the manuscript and provide valuable insights for clinical practice.

## Ethical approval

Not applicable.

## Consent

Not applicable.

## Sources of funding

Not applicable.

## Author contribution

All authors read and approved the final version of the manuscript.

## Conflicts of interest disclosure

The authors declare no conflicts of interest.

## Research registration unique identifying number (UIN)

1. Name of the registry: not applicable.
2. Unique identifying number or registration ID: not applicable.
3. Hyperlink to your specific registration (must be publicly accessible and will be checked): not applicable.

## Guarantor

Chao Su.

## Data availability statement

This manuscript is a comment. Don’t need a Data availability statement. However, all the data from the current study are publicly available.

## Provenance and peer review

Not applicable.
